# Distinguishing Parkinson’s Disease with GLCM Features from the Hankelization of EEG Signals

**DOI:** 10.3390/diagnostics13101769

**Published:** 2023-05-17

**Authors:** Mehmet Fatih Karakaş, Fatma Latifoğlu

**Affiliations:** 1 Faculty of Engineering and Architecture, Department of Biomedical Engineering, Erzincan Binali Yildirim University, Erzincan 24002, Turkey; 2 Faculty of Engineering, Department of Biomedical Engineering, Erciyes University, Kayseri 38280, Turkey

**Keywords:** EEG, Parkinson’s disease, diagnosis, classifier, Hankel matrix, GLCM features

## Abstract

This study proposes a novel method that uses electroencephalography (EEG) signals to classify Parkinson’s Disease (PD) and demographically matched healthy control groups. The method utilizes the reduced beta activity and amplitude decrease in EEG signals that are associated with PD. The study involved 61 PD patients and 61 demographically matched controls groups, and EEG signals were recorded in various conditions (eyes closed, eyes open, eyes both open and closed, on-drug, off-drug) from three publicly available EEG data sources (New Mexico, Iowa, and Turku). The preprocessed EEG signals were classified using features obtained from gray-level co-occurrence matrix (GLCM) features through the Hankelization of EEG signals. The performance of classifiers with these novel features was evaluated using extensive cross-validations (CV) and leave-one-out cross-validation (LOOCV) schemes. This method under 10 × 10 fold CV, the method was able to differentiate PD groups from healthy control groups using a support vector machine (SVM) with an accuracy of 92.4 ± 0.01, 85.7 ± 0.02, and 77.1 ± 0.06 for New Mexico, Iowa, and Turku datasets, respectively. After a head-to-head comparison with state-of-the-art methods, this study showed an increase in the classification of PD and controls.

## 1. Introduction

More than 200 years have passed since James Parkinson’s seminal article was published and, during that time, understanding of this disease has grown substantially [1]. Parkinson’s Disease (PD) is the second most common neurodegenerative disorder after Alzheimer’s, with an increasing prevalence with age, affecting approximately 2–3% of people over 65 years of age [2,3]. According to the World Health Organization (WHO) [4] and the Parkinson’s Foundation [5], the number of PD has doubled in the last 25 years, with over 10 million individuals with PD globally. 

PD is a degenerative process that affects primarily the substantia nigra which is a structure of the basal ganglia and other brainstem-pigmented neurons. The neurons afflicted with PD are unable to release dopamine which is a messenger substance between the brain and the rest of the body. As a result, both motor and non-motor symptoms develop, including mild tremor, changes in posture, walking, and facial expressions. Over time, these symptoms progress to loss of balance and slowness of movements, frequent falls, stiffness, hallucinations and delusions, mood and sleep disturbances, and cognitive failures [6]. The main clinical manifestations of PD are resting tremor, bradykinesia, rigidity, and postural reflex dysfunction [7]. 

The accuracy of clinical diagnoses, especially in the early stages of PD, has not significantly improved for about 25 years, and the average accuracy of 20 studies is around 80.6%. Correct diagnosis of PD is an essential requirement for patient counseling and therapy management [8]. Currently, the diagnosis of PD relies on the medical observation and clinical examination of patients by a specialist. Patients are examined according to some clinical diagnostic criteria, for example, the Unified Parkinson’s Disease Rating Scale (UPDRS). However, this conventional diagnostic method can be biased by a clinician and can lead to misclassification. Furthermore, early symptoms of PD are often mild and unnoticed; therefore, it is difficult to accurately diagnose, especially in the early stages of PD [9].

Various imaging methods can be used in the diagnosis of PD, depending on the type of parkinsonism. There are multiple types of parkinsonism such as idiopathic, vascular, drug-induced, and some other types. The most common type is idiopathic and it is also known as Parkinson’s Disease (PD) and, in this study, we dealt with this type of parkinsonism which means the cause is unknown [10]. Neuromelanin Magnetic Resonance Imaging (NM-MRI) can detect changes in the brain structure associated with PD, such as loss of dopaminergic neurons in the substantia nigra [11], while positron emission tomography (PET) and single-photon emission computed tomography (SPECT) can sensitively detect dopamine deficiency for diagnosing PD. However, these imaging techniques are expensive and require specialized equipment, limiting their widespread use for routine clinical diagnosis [12]. Other than imaging methods, the olfactory dysfunction test, which affects 90% of patients, can be used as an early clinical manifestation of PD [13]. Biomarker-based approaches involve the measurement of biological markers in blood, cerebrospinal fluid (CSF), or other bodily fluids that can provide information about disease presence and severity [14]. EEG has also been explored as a potential diagnostic tool for PD. As a non-invasive method, EEG-based methods offer several advantages over other diagnostic techniques, including low cost, non-invasiveness, and high temporal resolution. The number of EEG-based studies is increasing day by day [15,16]. 

EEG signals can be used to differentiate PD patients from healthy controls or patients with other movement disorders. EEG signals are inherently complicated and nonlinear. As a result, most linear feature extraction algorithms cannot be applied correctly to EEG signals because these algorithms do not work well for these kinds of signals [6]. EEG features such as reduced beta activity, non-linear features, statistical features, decrease in amplitude or changes in spectral power have been associated with the presence and severity of motor symptoms in PD, and machine learning algorithms have been developed to classify EEG signals based on these features [17,18,19,20]. 

A novel method is proposed for the diagnosis of PD detection in this study. In this study, we have especially used the reduced beta activity and the relative amplitude decrease in EEG signals, characteristics associated with PD. The method we use is performed by extracting Haralick GLCM features [21] from the Hankel matrix [22] of segmented EEG signals, which were first introduced for PD diagnosis. With this method, classifications can be made from very short-term EEG signal recordings. 

The main contributions of this study to the literature can be listed as follows: A proposed method for classifying PD using a large dataset that is publicly available and frequently used has been tested. A novel feature vector has been created by using GLCM features of the image obtained from the data matrix through the Hankelization of EEG signals. A high-performance classification method for PD has been presented by applying feature selection and machine learning methods to the obtained innovative feature set.

## 2. Materials and Methods

Datasets, preprocessing, feature extraction, and classification methods used for the proposed method are explained in this section. respectively. All analyses were performed on a computer using Windows-10 operating system, 3 GHz AMD Ryzen 5 4600H processor, 8 GB 3200 MHz RAM, and 4 GB NVDIA GeForce GTX 1650 graphics card. For reproducibility, all MATLAB codes had been run with the default random number generator as the initial condition.

### 2.1. Datasets

In this study, three publicly available datasets were used to classify PD. Electrode positions were obtained in the standard 10–20 EEG layout and from 64 channels with sintered Ag/AgCl electrodes at a 500 Hz sampling frequency for all datasets. 

The first data source is from the University of New Mexico [15,23]. Hereinafter, this data source will be named UNM. UNM contains records from a total of 54 patients, comprising 27 PD patients and 27 control groups. There are two session records for each patient in the PD group, taken 7 days apart. These were recorded while on the drug and 12 h after the last dose of dopaminergic medication for all individuals; thereby, there are two types of records: eyes closed for 1 min and eyes open for 1 min. 

The second data source is from the University of Iowa [15,23]. Hereinafter, this data source will be named UI. UI contains records from a total of 28 patients, comprising 14 PD patients and 14 control groups. These records were collected only with eyes open and on-drug. 

The third data source is from the University of Turku [24]. Hereinafter, this data source will be named UT. UT contains records from a total of 40 patients, comprising 20 PD patients and 20 control groups. These records were collected in both eyes-open and eyes-closed sessions while on-drug. 

Based on these three sources of records, seven different variants were created. Detailed information is provided in Table 1.

In all 3 data sources, the PD Control group was demographically matched with the PD group in terms of age and gender. Subjects did not differ in any measurements of education or premorbid intelligence. Detailed information about data sources is given in Table 2.

### 2.2. Preprocessing

**Figure 1:**
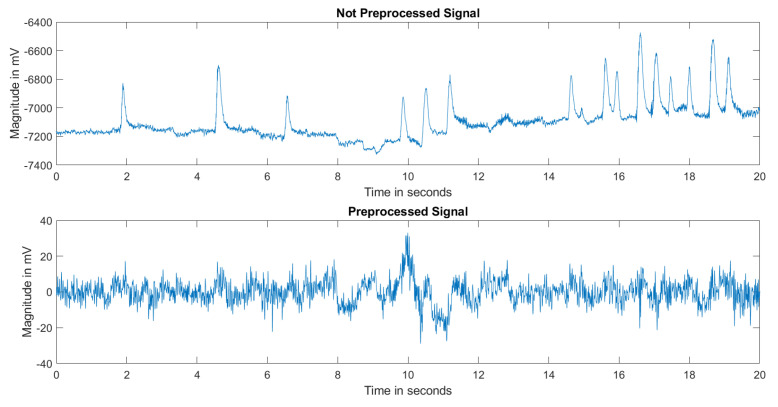
Comparison for not preprocessed and preprocessed signal.

First, all the records were organized to facilitate processing in the MATLAB 2022a (MathWorks, Inc., Natick, MA, USA) environment and to make them easily applicable for analysis. The organized records were preprocessed using EEGLAB in MATLAB [25]. Only channels that were common to all records, a total of 57 channels, were used for analysis. Other channels were excluded. The common channel information can be found in the Supplementary Materials, Figure S1 for the layout of EEG electrodes and Table S1 for channel names. All the records were filtered with a 1–50 Hz band pass filter. Afterward, the channel averages were re-referenced to the records. To remove noises from the records such as muscle, eye, heart, and line noise, among others, that fall between 0.9–1 among the signals, independent component analysis (ICA) [26] was performed. This process left only components related to brain activity in the records. As an example, Figure 1 shows the differences between the preprocessed and not preprocessed EEG records, with baseline shift, sudden and large peaks, and non-normalized structure. The preprocessing operations eliminated all these issues. 

### 2.3. Feature Extraction and Selection

**Figure 2:**
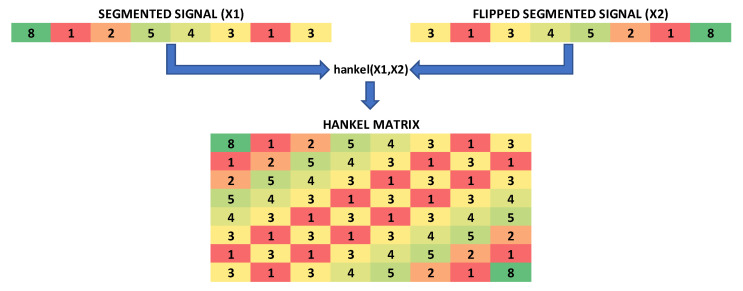
Hankelization process.

In the feature extraction process, the channels in the records of each subject are segmented into a total of 50 windows determined experimentally. Afterward, segmented data in the windows was transformed into a picture by making it 2D with a Hankel matrix as a novel approach. Hankel matrix is created by MATLAB’s Hankel function. In this function, a Hankel matrix of a vector signal such as an EEG record is defined as, firstly, a segmented EEG signal (X1) flipped left to right (X2). The first column of a Hankel matrix is X1 and the following columns are produced by shifting X1 one position at a time to the left, and in doing this the upper triangular matrix is formed. The last row of a Hankel matrix is X2 and the following rows are produced, shifting X2 one position at a time to the right and in doing this the lower triangular matrix is formed. Details are given in Figure 2. 

**Figure 3:**
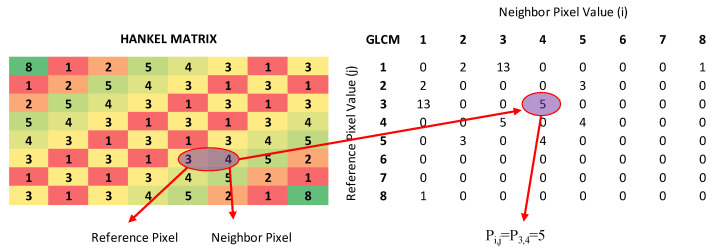
Generating GLCM matrix.

Using this Hankel matrix, GLCM was created by MATLAB’s graycomatrix function. This function: calculates how often the gray-level intensity of the pixel of interest in an image occurs with the gray-level intensity of the specified neighboring pixel. First, the Hankel matrix’s number of gray levels is scaled to 8 levels with MATLAB’s default settings. GLCM matrix is created by calculating over the scaled matrix [21,27]. Details are given in Figure 3.

Haralick texture features were extracted from each segment using the GLCM and a total of 19 features were obtained for each segment [21,28,29,30]. Brynolfsson’s MATLAB code was used to obtain these features [31] and notations for computing Haralick texture features and GLCM features are provided in Supplementary Materials in Tables S2 and S3.

The features obtained from each channel’s segment were converted into a vector. Here, for each channel, a total of 50 windows and 19 features were extracted with a length of 50 × 19, which is a total of 950 features. Each EEG recording of each subject consisted of 57 channels, resulting in a total of 950 features, and 57 channels were extracted with a length of 57 × 950, which is a total of 54,150 features for each subject.

To reduce the number of features in the analysis, a feature selection method was applied using the Chi-Square (chi2) method [32,33], which uses a statistical moment to select features that make sense for a particular model. The chi2 test was used to assess the relative variances of two distributions and determine which features depend on the output class label the most. The chi2 value for each feature in the dataset was calculated, and all features were sorted using their chi2 values in decreasing order. The first 100, 250, 500, 1000, 2500, and 5000 features with the highest scores from the chi2 method were empirically selected for the classification process.

### 2.4. Classification

By using the extracted and selected features, 10 × 5-fold and 10 × 10-fold cross-validation classification was performed for feedforward network (FF), support vector machine (SVM), and k-nearest neighbor (KNN) classifiers in MATLAB default settings. 

#### 2.4.1. Feed-Forward Network

Many classification problems are not linearly separable. We can separate the classes in such nonlinear problems by introducing more hyperplanes, or threshold units. This is usually done by adding an extra layer of threshold units, each of which does a partial classification of the input and sends its output to a final layer, which assembles partial classifications into a final classification. Such a network is called a multi-layer perceptron or feed-forward network (FF) [34].

#### 2.4.2. Support Vector Machine

Support vector machine (SVM) is a machine learning algorithm (MLA) that minimizes risk by offering different solutions to a number of linear and nonlinear problems. It is used to solve binary classification problems and can also be applied to multiclass classification problems. SVM is divided into two groups: linear SVM and nonlinear SVM, according to the state of the data. SVM is an effective learning algorithm in complex datasets, identifying patterns that are difficult to analyze [35].

#### 2.4.3. K-Nearest Neighbor

K-nearest neighbor is a classification algorithm that uses learning data that is the closest distance or most common object characteristics to classify objects. There are several steps involved in calculating K-NN, including determining the value of K and calculating Euclidean distance for each object from training data. Arranges objects into groups whose smallest Euclidean distance is determined by finding the minimum value of these distances among all objects in the group [36].

The default settings of these classifiers used in this study are given in Table 3. The aim of this study is to determine which classifier provides the best results among others. 

Additionally, the success of the classifier was examined by using the leave-one-out cross-validation (LOOCV) method for each dataset.

#### 2.4.4. Cross-Validation

Next, 5- and 10-fold cross-validation was used to evaluate the performance of the mode, respectively. The process of this verification method is described as follows: first, the entire dataset will be randomly divided into 5 and 10 copies; then, a single subset is randomly selected and retained as validation data to test the model, while the remaining 4 and 9 are used as training data to train the prediction model. This process is carried out 5 and 10 times; that is, every piece of data will be used as test data. Finally, the 5 and 10 results are averaged to obtain the final prediction [37].

#### 2.4.5. Leave One out Cross-Validation

The success of the classifiers was examined by this method. In this method, data are segmented based on the subjects: one subject for testing and the other remaining for training. This process is repeated until each subject has been used for the test [38].

### 2.5. Performance Parameters

To evaluate the performance of these models, the area under the receiver operating characteristics (ROC) curve (AUC), accuracy (ACC), sensitivity (SENS), specificity (SPEC), positive predictive value (PPV), and negative predictive values (NPV) were calculated. 

AUC is a performance measure that quantifies how well a binary classifier can distinguish between two classes. A value of 1 indicates a perfect classifier, while a value of 0.5 indicates a completely random classifier. ACC measures the proportion of correctly classified instances among all instances. SENS measures the proportion of true positive instances among all actual positive instances. SPEC measures the proportion of true negative instances among all actual negative instances. PPV measures the proportion of true positive instances among all instances that the classifier predicted as positive. NPV measures the proportion of true negative instances among all instances that the classifier predicted as negative.

## 3. Results

This study proposed a method based on the Hankelization and GLCM features to distinguish the PD group from the demographically matched control group. The method was validated using seven variants of datasets (UNM_All, UNM_Closed, UNM_Open, UNM_Off, UI, UT_Closed, and UT_Open) from three different data sources (UNM, UI, and UT). In this study, different success rates were obtained in the classifications.

Overall, the SVM method with 10 × 10 fold cross-validation using 1000 features yielded the best classification performance among other classifiers. However, some datasets showed better results using other classification methods. The results obtained in the SVM method with 10 × 10 fold cross-validation using 1000 features are presented in Table 4. When Table 4 is examined, the best results for 10 × 10 fold CV SVM were 94.9% AUC from UNM_Open, 92.41% ACC from UNM_All, 94.29% SENS from UI, 91.85% SPEC from UNM_All, 91.96% PPV from UNM_All, and 92.96% NPV from UNM_All. For UNM data source UNM_All dataset results; since UI data source only has one variant, UI dataset results; for UT data source, UT_Closed dataset; yielded better results according to Table 4 in terms of accuracy.

The classification performance results for the 10 × LOOCV SVM method used to validate the results are presented in Table 5. When Table 5 is examined the 10 × 10 fold CV SVM and 10 × LOOCV SVM methods had very similar results for all datasets, obtained with accuracies of 93.7% for UNM_All, 89.26% for UNM_Closed, 87.04% for UNM_Open, 83.33% for UNM_Off, 82.5% for UI, 76.92% for UT_Closed, and 59.75% for UT_Open.

A comparison of the classification performance with previous state-of-the-art methods is presented in Table 6. In Table 6, the proposed method is given as the 10 × 10 LOOCV SVM method in order to make a general comparison to the literature. All other performance results of classifiers are given in the Supplementary Material.

## 4. Discussion

We have designed and developed a novel method for the classification of PD from healthy and demographically matched control groups using GLCM features obtained from the Hankelization of EEG signals, which involves projecting a one-dimensional (1D) signal into a two-dimensional (2D) picture. Our proposed method is capable of and can achieve high accuracies in PD detection for these datasets and offers several advantages compared to previous methods reported in the literature. 

Firstly, we achieved an increase of approximately 10% in accuracy compared to previous methods. Some researchers have reported moderate accuracy results such as 88.51% by Shah [39], 69.2% by Sugden [43], 85.4% by Anjum et al. [15], 78% by Chaturverdi et al. [42], and 88.5% by Aljalal et al. [48]. Other researchers reported quite high accuracy results, such as 99.2% by Lee et al. [50], 94.1% by Sugden [43], 99.58% and 99.41% by Aljalal [48,51], 94.3% by Vannesta [45], and 99.62% by Yuvaraj [46]. However, some studies in the literature reported have had issues, such as data leakage from the training dataset to the test dataset, unbalanced classes, demographically unmatched groups, or artificially replicated EEG records, leading to very high accuracies. In our study, we have addressed these issues by splitting the train and test dataset without any data leakage, using short EEG records, and ensuring demographically matched groups, without artificially replicating EEG records, and still achieved quite high accuracies.

Secondly, while some previous studies [42,45,46] only performed one round of cross-validation, we confirmed the classification of the data by performing 10 rounds of cross-validation and LOOCV.

Thirdly, we did not tune the optimization parameters of the classifiers and instead left them at default settings. While tuning these parameters could potentially yield better results, problems such as overfitting could become an issue.

Fourthly, we observed that our method can achieve good results on both drug and off-drug sessions.

Finally, our feature extraction approach, which involves transforming the signal into a picture using Hankelization and obtaining Haralick-GLCM features from EEG signals, has not been used before in the literature and is, therefore, a very novel method. The gold-standard diagnosis of neurodegenerative diseases such as PD requires post-mortem assessment. Clinical vs. pathology-confirmed diagnoses are approximately 90% accurate and results are very close in terms of the accuracy reported here.

However, this method has some limitations that need to be addressed in future studies.

Firstly, the method is time-consuming. Increasing the number of windows in the method reduces the number of data to be segmented. In this case, the number of extracted features is becoming quite high. Reducing the number of windows increases the amount of data to be segmented. In this case, the size of the segmented images obtained as a result of the Hankelization process of the EEG segment increases considerably. In both conditions, the processing load increases, and this causes an enhancement in the time taken for feature extraction. In our studies, we have obtained the best result in a feature number of 1000 and standard 50 windows for acceptable time experimentally. Instead of extracting a large number of features in the first place and then selecting features from these features, directly extracting a small number of features and giving these features to the classifier probably will give faster results.

Secondly, channel selection can be performed to reduce the number of features to be extracted, making the method work faster.

Finally, in future studies, separating the EEG signals into sub-bands instead of filtering the EEG signals globally between 1–50 Hz, may give better results for distinguishing PD and control groups. In conclusion, our proposed method showed promising results for the classification of PD from healthy and demographically matched control groups, and it has several advantages compared to previous methods with these datasets. Moreover, as an EEG-based classifier, it is low-cost, easily accessible, and widely applicable compared to other methods. Future work with a large sample will demonstrate the true generalizability of this method.

## Figures and Tables

**Table 1:** Data sources and variants with conditions.

Data Source	Dataset	Eyes Condition	Drug Condition
UNM	UNM_ALL	Open/Closed	On
	UNM_OPEN	Open	On
	UNM_CLOSED	Closed	On
	UNM_OFF	Open/Closed	Off
UI	UI	Open/Closed	On
UT	UT_OPEN	Open	Off
	UT_CLOSED	Closed	Off

**Table 2:** Parkinson’s Disease and control participant demographics.

(Mean ± STD)	UNM		UI		UT	
Condition	PD	Control	PD	Control	PD	Control
Sex	17 M/10 F	17 M/10 F	6 M/8 F	6 M/8 F	9 M/11 F	8 M/12 F
Age	69.5 ± 8.7	69.5 ± 9.3	70.5 ± 8.7	70.5 ± 8.7	69.8 ± 7.2	67.8 ± 6.2
MMSE	28.7 ± 1	28.8 ± 1	-	-	27.8 ± 1.8	28.2 ± 1.5
MOCA	-	-	25.9 ± 2.7	27.2 ± 1.7	-	-
UPDRS	22.2 ± 10.3	-	13.4 ± 6.6	-	28.9 ± 16.4	5.1 ± 3.5
Years from Diagnosis	5.7 ± 4.2	-	5.6 ± 3.2	-	6.4(4.9)	-
Recording Minute	3.59 ± 1	3.63 ± 1.8	3.11 ± 1.2	3.17 ± 0.9	2.55 ± 0.06	2.51 ± 0.2
BDI	7.6 ± 5.3	4.8 ± 4.8	-	-	8.4 ± 6.2	5.0 ± 3.0
LED	707.4 ± 448.6	-	796 ± 409	-	663.2 ± 509.1	-
NAART	45.2 ± 10.3	47.1 ± 7.5	-	-	-	-

Abbreviations: M = male; F = female; MMSE = Mini Mental State Exam; MOCA = Montreal Cognitive Assessment; UPDRS = United Parkinson’s Disease Rating Scale (motor); BDI = Beck’s Depression Inventory; LED = L-Dopa equivalence dose in mg.

**Table 3:** Default settings for classifiers.

FF	SVM	KNN
Layer Size = [10] Activation Function = Relu	Kernel Function = Linear Kernel Scale = 1 Box Constraint = 1	1 Neighbor Euclidean Distance

**Table 4:** Classification performance results for 10 × 10-fold cross-validation SVM.

	AUC	ACC	SENS	SPEC	PPV	NPV
UNM_All	92.84	92.41	92.96	91.85	91.96	92.96
	(91.08–94.24)	(90.74–94.44)	(88.89–96.3)	(88.89–92.59)	(89.29–92.86)	(89.29–96.15)
UNM_Closed	94.44	89.07	90	88.15	88.38	89.84
	(92.18–95.47)	(87.04–90.74)	(88.89–92.59)	(85.19–88.89)	(85.71–89.29)	(88.46–92.31)
UNM_Open	94.9	89.44	89.63	89.26	89.46	89.66
	(92.87–95.61)	(87.04–94.44)	(85.19–92.59)	(85.19–96.3)	(85.71–96.15)	(85.71–92.86)
UNM_Off	90.66	83.89	88.15	79.63	81.34	87.16
	(87.93–92.46)	(79.63–87.04)	(81.48–92.59)	(70.37–85.19)	(75.76–85.71)	(80.77–91.67)
UI	87.4	85.71	94.29	77.14	80.5	93.31
	(82.14–89.8)	(82.14–89.29)	(85.71–100)	(71.43–78.57)	(76.47–82.35)	(84.62–100)
UT_Closed	84	77.18	73	81.58	80.86	74.45
	(77.37–88.95)	(66.67–84.62)	(60–85)	(68.42–89.47)	(70–88.89)	(63.64–84.21)
UT_Open	67.85	63.25	77	49.5	60.49	68.26
	(64.75–71.25)	(60–67.5)	(70–80)	(40–55)	(57.14–64)	(62.5–73.33)

**Table 5:** Classification performance results for 10 × LOOCV SVM.

	AUC	ACC	SENS	SPEC	PPV	NPV
UNM_All	94.39	93.7	93.22	94.16	94.16	93.51
	(87.72–100)	(90.74–98.15)	(88–100)	(88.89–100)	(88.46–100)	(88.46–100)
UNM_Closed	94.11	89.26	92.22	86.23	87.26	91.68
	(89.71–96.98)	(83.33–92.59)	(88–96.15)	(74.07–96)	(78.13–96.3)	(88.89–96.67)
UNM_Open	94.55	87.04	86.23	87.76	86.88	87.23
	(90.67–98.32)	(77.78–92.59)	(72–96.3)	(81.25–95.83)	(72.73–96.43)	(78.79–96)
UNM_Off	91.07	83.33	87.99	78.53	80.45	87.29
	(83.68–95.45)	(72.22–88.89)	(74.07–96.3)	(70.37–90.63)	(71.43–88.89)	(73.08–95)
UI	85.21	82.5	88.9	75.35	79.42	85.82
	(78.06–96.11)	(75–92.86)	(78.57–100)	(57.14–94.44)	(66.67–93.75)	(71.43–100)
UT_Closed	83.21	76.92	73.97	80.65	80.57	73.07
	(72.86–91.3)	(66.67–84.62)	(64–85.71)	(70–93.75)	(66.67–94.12)	(52.63–90.48)
UT_Open	61.93	59.75	78.61	41.53	55.29	68.31
	(39.64–80.3)	(45–75)	(47.06–89.47)	(23.53–68.18)	(38.1–68.18)	(50–83.33)

**Table 6:** Comparison with previous methods.

UNM_All		UI		UT_Closed	
Shah et al. [39]	88.5	Qiu et al. [9]	96.31	Kurbatskaya et al. [40]	82.2
Anjum et al. [15]	85.2	Anjum et al. [15]	85.7	Suuronen et al. [41]	76
Chaturverdi et al. [42]	72.2	Sugden et al. [43]	83.8	Shabanpour et al. [44]	63.44
Vanneste et al. [45]	72.2	Proposed	85.71	Proposed	76.92
Yuvaraj et al. [46]	59.3				
Lee et al. [47]	89.3				
Sugden et al. [43]	69.2				
Aljalal et al. [48]	87.04				
Avvaru et al. [49]	79.25				
Proposed	93.7				

## Data Availability

All datasets used are publicly available datasets and are available for download from the links below: the University of New Mexico Dataset: http://predict.cs.unm.edu/downloads.php d013 dataset (accessed on 4 March 2023); the University of Iowa Dataset: http://predict.cs.unm.edu/downloads.php d013 dataset (accessed on 4 March 2023) and the University of Turku Dataset: https://osf.io/pehj9/ (accessed on 4 March 2023).

## Supplementary Materials

The following supporting information can be downloaded at: https://www.mdpi.com/article/10.3390/diagnostics13101769/s1, Figure S1: Common Electrode Locations; Table S1: Common Channel Names List; Table S2: Notations to compute Haralick Texture Features. All performance parameters and evaluation results in this article are given in xls format; Table S3: GLCM Texture Features.
