# Supplementary material for: Distinguishing Parkinson’s Disease with GLCM Features from the Hankelization of EEG Signals
Source: Diagnostics (Basel). 2023 May 17;13(10):1769. doi: 10.3390/diagnostics13101769 (PMC10216898; doi:10.3390/diagnostics13101769)
Supplement: Supplementary file 1 [file diagnostics-13-01769-s001.zip › supplementary.pdf]

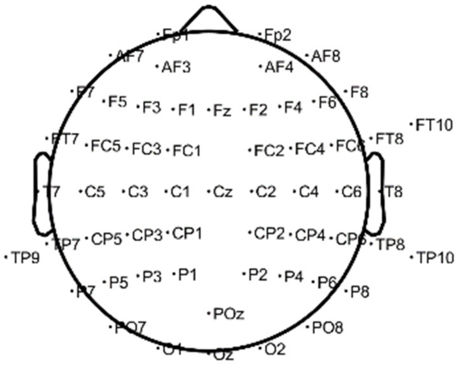

Figure S1. Common Electrode Locations.

Table S1. Common Channel Names List

{'AF3','AF4','AF7','AF8','C1','C2','C3','C4','C5','C6','CP1','CP2','CP3','CP4','CP5','CP6','Cz','F1','F2','F3','F4','F5','F6','F7','F8','FC1','FC2','FC3','FC4','FC5','FC6','Fp1','Fp2','FT10','FT7','FT8','Fz','O1','O2','Oz','P1','P2','P3','P4','P5','P6','P7','P8','PO7','PO8','POz','T7','T8','TP10','TP7','TP8','TP9'}

Table S2. Notations to compute Haralick Texture Features

Notation	Definition
$x(i,j)$	Element i, j in the unnormalized GLCM
N	Number of gray-levels
$P(i,j)$	$\frac{x(i,j)}{\sum_{i=1}^{N}\sum_{j=1}^{N} x(i,j)}$
$P_x(i)$	$\sum_{j=1}^{N} P(i,j)$
$P_y(j)$	$\sum_{i=1}^{N} P(i,j)$
μ_x	$\sum_{i=1}^{N} iP_x(i)$
μ_y	$\sum_{j=1}^{N} jP_y(j)$
μ	For symmetric GLCM $\mu_x = \mu_y = \mu$
${\sigma_x}^2$	$\sum_{i=1}^{N} (i-\mu_x){P_x}^2(i)$
${\sigma_y}^2$	$\sum_{j=1}^{N} (j-\mu_y){P_y}^2(j)$
$P_{x+y}(k)$	$\sum_{i=1}^{N}\sum_{j=1}^{N} P(i,j)\,, i+j=k$

$P_{x-y}(k)$	$\sum_{i=1}^{N}\sum_{j=1}^{N} P(i,j)\,, \lvert i-j \rvert = k$
μ_{x+y}	$\sum_{k=2}^{2N} kP_{x+y}(k)$
μ_{x-y}	$\sum_{k=0}^{N-1} kP_{x-y}(k)$
HX	$-\sum_{i=1}^{N} P_x(i) log(P_x(i))$
HY	$-\sum_{i=1}^{N} P_y(i) log(P_y(i))$
HXY	$-\sum_{i=1}^{N}\sum_{j=1}^{N} P(i,j) log(P(i,j))$
$HXY1$	$-\sum_{i=1}^{N}\sum_{j=1}^{N} P(i,j) log[P_x(i)P_y(j)]$
$HXY2$	$-\sum_{i=1}^{N}\sum_{j=1}^{N} P_x(i)P_y(j) log[P_x(i)P_y(j)]$

Table S3. GLCM Texture Features

**Feature**	**Expression**
Autocorrelation [1]	$AUTOC = \sum_{i=1}^{N}\sum_{j=1}^{N}(i\ x\ j)P(i,j)$
Cluster Prominence [2]	$PROM = \sum_{i=1}^{N}\sum_{j=1}^{N}\{i+j-2\mu\}^3 P(i,j)$
Cluster Shade [2]	$SHADE = \sum_{i=1}^{N}\sum_{j=1}^{N}\{i+j-2\mu\}^4 P(i,j)$
Contrast [2]	$CONTRAST = \sum_{i=1}^{N}\sum_{i=1}^{N}(i-j)^2 P(i,j)$
Correlation [2]	$CORR = \sum_{i=1}^{N}\sum_{j=1}^{N}\left(\frac{i-\mu_x}{\sigma_x}\right)\left(\frac{j-\mu_y}{\sigma_y}\right)P(i,j)$
Difference entropy [2]	$DENT = -\sum_{k=0}^{N-1} P_{x-y}(k) \log\,(P_{x-y}(k))$
Difference variance [2]	$DVAR = \sum_{k=0}^{N-1}\left(k-\mu_{x-y}\right)^2 P_{x-y}(k)$
Dissimilarity [1]	$DISSIMILARITY = \sum_{i=1}^{N}\sum_{j=1}^{N}\lvert(i-j)\rvert P(i,j)$
Energy [2]	$ENR = \sum_{i=1}^{N}\sum_{i=1}^{N} P(i,j)^2$
Entropy [2]	$ENT = -\sum_{i=1}^{N}\sum_{j=1}^{N} P(i,j)\ x\ log(P(i,j))$

Homogeneity [1]	$HOM = \sum_{i=1}^{N}\sum_{j=1}^{N}\frac{P(i,j)}{1+(i-j)^2}$
Informational measure of correlation1 [2]	$IMC1 = \frac{HXY - HXY1}{\max(HX,HY)}$
Informational measure of correlation2 [2]	$IMC2 = \sqrt{1-\exp(-2(HXY2-HXY))}$
Inverse difference [3]	$IDM = \sum_{i=1}^{N}\sum_{i=1}^{N}\frac{P(i,j)}{1+\lvert i-j\rvert}$
Maximum probability [1]	$PROB = Max_{i,j}(P(i,j))$
Sum average [2]	$SAVG = \sum_{k=2}^{2N} kP_{x+y}(k)$
Sum entropy [2]	$SENT = -\sum_{k=2}^{2N} P_{x+y}(k)\log(P_{x+y}(k))$
Sum of squares: Variance[2]	$SQVAR = \sum_{i=1}^{N}\sum_{j=1}^{N}(k-\mu)^2P(i,j)$
Sum variance[2]	$SVAR = \sum_{k=2}^{2N}(k-\mu_{x+y})^2P_{x+y}(k)$

1. Soh, L-K, and Costas Tsatsoulis. "Texture Analysis of Sar Sea Ice Imagery Using Gray Level Co-Occurrence Matrices." *IEEE Transactions on geoscience and remote sensing* 37, no. 2 (1999): 780-95.
2. Haralick, Robert M, Karthikeyan Shanmugam, and Its' Hak Dinstein. "Textural Features for Image Classification." *IEEE Transactions on systems, man, and cybernetics*, no. 6 (1973): 610-21.
3. Clausi, David A. "An Analysis of Co-Occurrence Texture Statistics as a Function of Grey Level Quantization." *Canadian Journal of remote sensing* 28, no. 1 (2002): 45-62.
